# A CTC-Based Speech Recognition Network Fusing Local Convolution and Global Attention

**DOI:** 10.3390/s26061865

**Published:** 2026-03-16

**Authors:** Huijuan Hu, Chenyang Tang, Ping Tan, He Xu

**Affiliations:** 1 School of Computer Science, Nanjing University of Posts and Telecommunications, Nanjing 210023, China; hhj@njupt.edu.cn (H.H.); 1024041005@njupt.edu.cn (C.T.); 2 School of Business, Tongda College of Nanjing University of Posts and Telecommunications, Yangzhou 225127, China; tanping5.20@njupt.edu.cn

**Keywords:** automatic speech recognition, wav2vec 2.0, information fusion, dual-branch architecture, task-aware gating, CTC alignment

## Abstract

**Highlights:**

**What are the main findings?**

- A dual-branch architecture (DBA) is proposed to decouple temporal modeling into parallel local convolutional and global attention streams.
- A task-aware gating mechanism is designed to adaptively fuse heterogeneous features based on acoustic confidence.

**What are the implications of the main findings?**

- The method resolves the conflict between the global smoothing of wav2vec 2.0 and the local discriminative needs of Connectionist Temporal Classification (CTC) alignment.
- The approach significantly improves robustness in fast-speech scenarios, achieving a 15.3% relative performance gain.

**Abstract:**

Integrating wav2vec 2.0 with Connectionist Temporal Classification (CTC) for automatic speech recognition (ASR) often involves a trade-off between capturing global semantic consistency and maintaining local feature discriminability. This study proposes DBA-wav2vec 2.0, an architecture designed to manage these modeling requirements by decoupling temporal modeling into parallel local and global streams at the encoder–decoder interface. Depthwise separable convolutions are utilized to capture local acoustic structures, while a self-attention path is retained for long-range dependencies. A task-aware gating mechanism is introduced to integrate these heterogeneous features. By adjusting fusion weights based on acoustic input characteristics, the gate facilitates the refinement of posterior probability distributions, leading to more distinct alignment points. Experimental results on AISHELL-1 and ST-CMDS datasets show relative Character Error Rate (CER) reductions of 6.4% and 7.4%, respectively, compared to a baseline wav2vec 2.0 model. Further evaluations under varying speaking rates demonstrate a 15.3% relative improvement in fast-speech scenarios, suggesting that structural adaptation at the decoding interface can enhance the robustness of CTC-based systems against temporal variations.

## 1. Introduction

Automatic Speech Recognition (ASR) aims to convert continuous acoustic signals into text sequences. Deep learning has driven a paradigm shift in research toward End-to-End (E2E) architectures, which have become the core approach for current ASR systems [1], significantly simplifying topologies while enhancing recognition accuracy [2]. Early hybrid architectures, such as the cascading of Hidden Markov Models (HMMs) and Gaussian Mixture Models (GMMs) [3], relied heavily on manual feature engineering and suffered from lengthy modeling due to segmented optimization. The subsequent introduction of Bi-directional Long Short-Term Memory (Bi-LSTM) networks improved acoustic representation [4] but remained prone to error accumulation. With the maturation of E2E technologies, methods like joint modeling have further strengthened the ability to handle complex tasks [5]. Among these, the Connectionist Temporal Classification (CTC) algorithm has become a cornerstone of sequence modeling due to its ability to function without explicit alignment annotations [6]. Subsequently, Deep Speech 2 [7], attention mechanisms [8], and the Listen, Attend, and Spell (LAS) framework [9] further expanded the boundaries of modeling long-sequence dependencies.

### 1.1. State of the Research Field

Recently, Self-Supervised Learning (SSL) has positioned models like wav2vec [10] and wav2vec 2.0 [11]—which enhance feature discrimination through masked contrastive learning—as core technologies in contemporary ASR. E2E ASR significantly simplifies system structure and training workflows compared to traditional Deep Neural Network-Hidden Markov Model (DNN-HMM) [12]. Subsequent studies, such as HuBERT [13], have further enhanced the semantic abstraction depth of features. Such models excel in capturing long-range context and maintaining semantic consistency, demonstrating strong generalization potential in multilingual scenarios like Cross-lingual Speech Representation (XLS-R) [14].

To balance short-term local structures and long-range semantic dependencies, the academic community has explored parallel or branched temporal modeling architectures. Approaches represented by Branchformer [15] and E-Branchformer [16] construct parallel branches within the encoder layer to extract local continuous features and global dependencies separately. Zipformer [17] improves representation efficacy through the reorganization of multi-scale resolutions. However, most existing methods focus on intra-layer feature fusion to build general efficient representations, rarely performing differentiated structural adaptation specifically for the decoding interface or alignment mechanisms.

### 1.2. Problem Statement and Contribution

Mechanistic analysis indicates that while the wav2vec 2.0 encoder focuses on utilizing global context to capture deep semantics, the attention smoothing effect of deep Transformers often leads to rank degeneration in the temporal dimension, weakening feature discriminability [18]. In contrast, the convergence of CTC is highly dependent on the local discriminability of input features to form precise alignment “spikes,” and its optimization process frequently faces challenges related to path search uncertainty [19]. This inductive bias conflict—between global semantic bias and the need for local alignment precision—often induces temporal shifts in predicted peaks or boundary blurring [20]. Moreover, under CTC constraints, high-level representations tend to coarsen as the receptive field expands, making it difficult to preserve precise time-step boundary information [21]. Although architectures like Conformer [22] attempt to alleviate such conflicts, they are limited by unified modeling approaches, especially under drastic speaking rate fluctuations [23]. Similarly, while Branchformer employs parallel branches, it relies on intra-layer fusion designed for training encoders from scratch. When such blocks are stacked as adapters on top of pre-trained transformers, their continued mixing of attention mechanisms tends to exacerbate the “smoothing” issue without explicitly restoring local boundaries. In contrast, our approach introduces a “late-stage decoupling” inductive bias.

We argue that under the CTC paradigm, relying on a single temporal modeling path makes it difficult to balance alignment stability with semantic discriminability. To address this, this paper proposes a dual-branch architecture (DBA) for the wav2vec 2.0 framework, referred to as DBA-wav2vec 2.0. This architecture deploys parallel local perception and global attention channels at the top of the pre-trained encoder to construct a functionally decoupled modeling topology. We utilize depthwise separable convolutions (inspired by QuartzNet [24]) for the local branch to capture phoneme-level boundaries, fitting the conditional independence assumption of CTC. Furthermore, a task-aware dynamic gating strategy is designed to adaptively adjust the weight ratio of local details to global context. Experimental results on AISHELL-1 and ST-CMDS datasets demonstrate that this approach significantly enhances the sharpness of the posterior probability distribution and improves robustness in complex scenarios.

The remainder of this paper is organized as follows: Section 2 details the proposed DBA-wav2vec 2.0 architecture, including the design of the local and global branches and the task-aware branch fusion strategy. Section 3 describes the experimental setup and evaluates the performance on public Mandarin datasets. Section 4 provides a comprehensive discussion on alignment stability, the effectiveness of the dual-branch structure, and the impact of deployment strategies. Finally, Section 5 summarizes the conclusions of this study.

## 2. Methods

### 2.1. Overall Architecture

In E2E ASR tasks, the wav2vec 2.0 pre-trained encoder, with its multi-layer Transformer architecture, performs unified and deep contextual modeling on input speech. This design endows the model with a strong capability to capture semantic consistency across time steps, which is highly beneficial for downstream tasks centered on semantic discrimination. However, when this architecture is placed under the CTC framework, its inherent inductive bias reveals limitations: the training process of the CTC algorithm relies heavily on searching for monotonic alignment paths between sparse effective labels and numerous blank frames. This mechanism places extremely high demands on the local discriminability, boundary sensitivity, and alignment stability of features in the temporal dimension. In other words, while the Transformer tends to generate smooth global semantic representations, CTC decoding urgently requires precise local acoustic boundaries, creating a significant structural tension between their modeling objectives.

**Figure 1:**
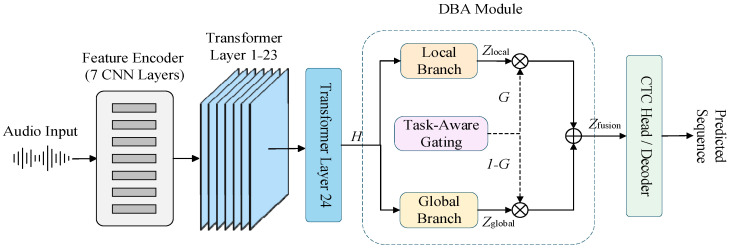
Overall architecture of the proposed DBA-wav2vec 2.0 model. The audio input is processed by a 7-layer CNN Feature Encoder and 24 Transformer layers. The DBA Module performs dynamic feature fusion using a Local Branch and a Global Branch. Solid arrows represent the primary data flow, while dashed arrows indicate the distribution of gating coefficients G and 1−G. Different colors are used to distinguish functional stages: feature encoding (grey and light blue), the DBA fusion module (orange and purple), and the CTC decoding stage (cyan).

To resolve this conflict between modeling objectives, this paper abandons the traditional unified temporal modeling path and instead introduces an explicit DBA at the high level of the wav2vec 2.0 encoder (as shown in Figure 1). Unlike the single data stream of the original architecture, this design strategically deploys the core reconstruction module (DBA Module) after the top-level Transformer Layer 24. This topological change aims to implement a “divide-and-conquer” strategy: at the critical node before features are fed into the CTC decoding head, utilizing the decoupling characteristics of parallel paths preserves the rich semantic information learned by the deep network while specifically reinforcing local representation precision through a dedicated structure.

Specifically, high-level features are diverted into two parallel paths with distinct functions: the Local Branch focuses on phoneme-level short-term continuous structures to enhance local separability between time steps, satisfying CTC’s need for alignment stability; the Global Branch continues to leverage the Transformer’s advantages, responsible for capturing long-range contextual dependencies to maintain semantic consistency. This design achieves an explicit separation of “alignment modeling” and “semantic modeling” tasks at the physical structural level within the CTC framework. To ensure the effective synergy of these two types of features, we further designed a Task-Aware Branch Fusion mechanism. This mechanism allows the model to adaptively adjust the weights of local and global information at different temporal positions according to the current alignment state and modeling needs. It is worth noting that since the dual-branch structure directly participates in the backpropagation of the CTC loss, the two paths are forced to learn differentiated temporal statistical properties during training. This division of labor cannot be equivalently achieved by simple module stacking or parameter weighting. Furthermore, probing studies on self-supervised models indicate that the top layers of encoders often tend to lose low-level acoustic details; thus, introducing a parallel local branch at the top provides an irreplaceable theoretical basis for compensating for acoustic alignment information.

In summary, the DBA-wav2vec 2.0 architecture proposes a temporal modeling reconstruction scheme oriented towards the CTC alignment mechanism. Unlike Conformer or Branchformer, which focus on fusing features within the encoder layers (intra-layer), this paper focuses on the systemic reconstruction at the decoding front-end, stripping and re-fusing functional roles in speech representation. The overall processing flow of the model is shown in Figure 1: the original audio is converted into low-level acoustic features via the Feature Encoder (composed of seven convolutional neural network (CNN) layers), then high-level representations are extracted through multi-layer Transformer encoding; on this basis, the dual-branch structure performs differentiated modeling of local and global features, which are finally sent to the CTC decoder after task-aware fusion. This approach fundamentally improves the model’s adaptability to CTC alignment stability while maintaining pre-trained semantic advantages.

### 2.2. Design of Local and Global Branches

**Figure 2:**
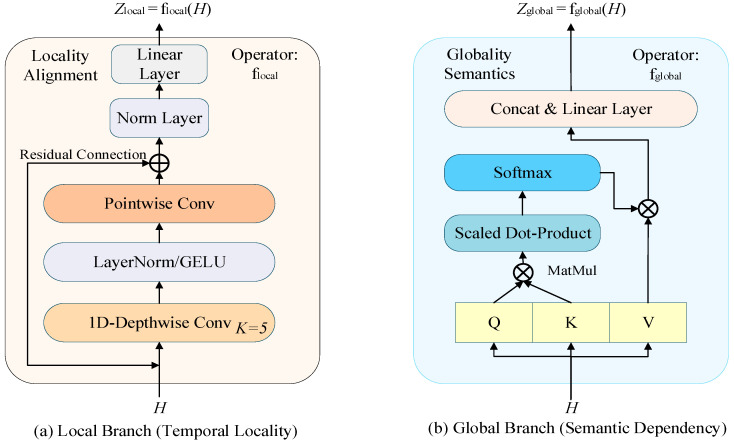
Detailed design of (**a**) the Local Branch and (**b**) the Global Branch. Functional modules are distinguished by colors: orange blocks represent convolutional layers, blue blocks denote attention-related operations, and yellow blocks represent Q, K, V matrices. Solid arrows indicate the data flow, while the curved arrow in (**a**) specifically represents the residual connection. Symbol ⊕ denotes element-wise addition and ⊗ denotes multiplication operations.

Speech signals naturally present multi-scale structural characteristics in the temporal dimension, which map to distinct modeling demands under the CTC architecture: short-term local context governs phoneme boundaries and time-step separability, serving as the cornerstone for stable alignment paths; whereas long-range context carries lexical and syntactic logic, directly determining the discriminative precision of the final recognition result. Based on this theoretical premise, we constructed an explicit dual-branch temporal modeling structure at the high level of the encoder to decouple and optimize these two mutually constraining objectives. The detailed design is shown in Figure 2.

Let the high-level feature sequence output by the wav2vec 2.0 encoder be:

(1) $$H=h_{1},h_{2},\ldots ,h_{T},\;h_{t}\in R^{d}$$

where T denotes the sequence length (number of time steps) and d represents the hidden dimension of the encoder features.

The Local Branch is used to characterize the local continuous structure in the temporal dimension, and its internal operator composition is shown in Figure 2a. We employ multi-layer 1D Depthwise Separable Convolutions to construct the local branch operator $f_{local}$. This convolution paradigm decouples spatial modeling from channel mapping and can capture strong temporal translation invariance features with an extremely low parameter load [25]. This design philosophy was first proven efficient in visual tasks [26]. Meanwhile, reasonable feature extraction and pooling operations are crucial for maintaining local discriminability when processing the temporal representation of acoustic features [27]. This operator reinforces boundary sensitivity and temporal separability between adjacent time steps through a fixed-size local receptive field (k=5). To ensure the effective flow of low-level acoustic details, this branch uses a Residual Connection (as shown on the left of the figure) for identity path mapping and utilizes a linear mapping layer to process the feature dimension to remain consistent with the encoder dimension d. Through this convolutional structural constraint, the model is forced to introduce spatial locality bias within the time step, making its modeling objective serve the local separability requirements during the time alignment process. Unlike generic blocks (e.g., Conformer) that sandwich convolutions between attention layers, our parallel design ensures that the local acoustic cues are not diluted by global mixing before reaching the fusion gate. This lightweight convolutional constraint has been proven to have higher inductive bias efficiency than the self-attention mechanism in capturing local phoneme-level acoustic patterns. Its output is represented as:

(2) $$Z_{local}=f_{local}\left(H\right)$$

The modeling goal of this branch is not semantic abstraction but rather serving the local separability requirements of the alignment process.

The Global Branch is used to model long-range dependencies across time steps. Its output is represented as:

(3) $$Z_{global}=f_{global}\left(H\right)$$

where $f_{global}$ denotes the global modeling function based on Multi-Head Self-Attention (MHSA), which is used to capture long-range contextual information and semantic consistency in the speech sequence [28]. This branch continues the semantic modeling inductive bias formed during the wav2vec 2.0 pre-training phase and is an important source of final recognition discriminability. As shown in Figure 2b, this branch captures long-range consistency through QKV mapping and Scaled Dot-Product Attention operators. By comparing subgraphs Figure 2a,b, it is evident that the two paths have significant mathematical complementarity, providing “micro-precision” and “macro-background” for the subsequent alignment task, respectively.

It must be emphasized that the proposed dual-branch architecture is not a simple stacking of parallel computations but rather achieves deep decoupling of “alignment stability modeling” and “semantic discriminability modeling” at the structural level. Unlike existing strategies of layer-by-layer fusion inside the encoder, we specifically deploy the branching module after the top-level output of the encoder. This strategic layout, under the constraint of the CTC loss function, forces different branches to learn differentiated temporal statistical characteristics, thereby establishing specific functional divisions for each branch in the physical structure.

### 2.3. Task-Aware Branch Fusion Strategy

**Figure 3:**
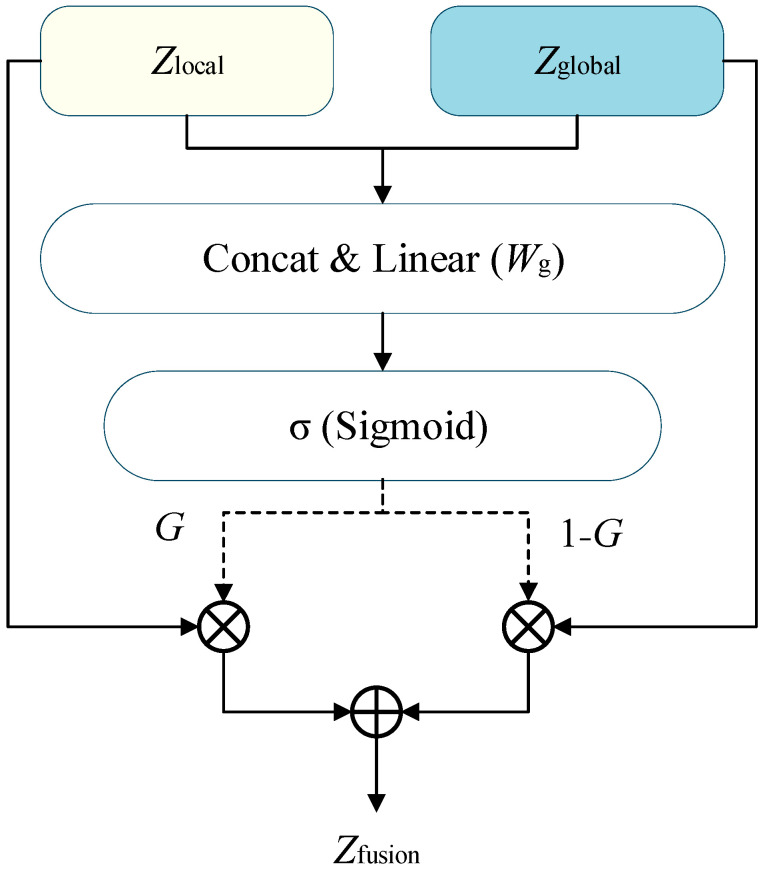
Diagram of the Task-Aware Gating Mechanism. The light yellow and light blue boxes represent the input features from the local and global branches, respectively. Solid arrows denote the primary flow of feature representations, while dashed arrows indicate the distribution of the calculated gating weights (G and 1−G). The symbols ⊗ and ⊕ represent element-wise multiplication and addition, respectively.

Under the CTC framework, the supervision signal in the temporal dimension exhibits high sparsity, leading to significant heterogeneity in the modeling demands of different time steps in the alignment path: critical time steps corresponding to effective labels or boundaries have strict requirements for local separability and alignment stability; while the vast majority of blank time steps rely more on contextual semantic consistency to maintain overall discriminability. Addressing this characteristic, we propose a Task-Aware Branch Fusion strategy, aiming to allow the model to adaptively balance the contribution weights of local and global branches on the time axis. The logic interaction and calculation flow of this mechanism are shown in Figure 3, where solid arrows depict the data path of feature tensors, and dashed arrows represent the generation and flow of gating control signals.

Research by Liu et al. [29] shows that effective fusion of multi-level acoustic features can significantly improve the representation capability of deep learning models for speech signals. Inspired by this, we employ a learnable gating mechanism to fuse the two branches. This design references the processing method of Gated Units in multi-source information fusion [30], formulated as:

(4) $$\begin{array}{cc} G & =\sigma \left(W_{g}\left[Z_{local};Z_{global}\right]+b_{g}\right)\\ Z_{fusion} & =G⊙Z_{local}+\left(1-G\right)⊙Z_{global} \end{array}$$

where σ represents the Sigmoid activation function, [⋅;⋅] denotes feature concatenation, $W_{g}\in R^{2d\times d}$ and $b_{g}\in R^{d}$ are learnable gating parameter matrices, $G\in {\left[0,1\right]}^{T\times d}$ is the dimension-wise gating weight matrix, and ⊙ denotes element-wise multiplication. This dynamic selection mechanism draws on the adaptive adjustment concept of Gated Linear Units (GLU) in long-sequence dependency modeling [31]. From the topological connection in Figure 3, it can be observed that the concatenated features are first input into the gate generation unit to learn the dimension-level weight G. Subsequently, a polarity shift occurs in the signal flow: the left path weight remains G, while the right path generates a complementary weight factor 1−G via a subtraction operator. These two complementary signals act on the outputs of the original local branch and global branch, respectively. This dynamic game mechanism not only ensures that $Z_{fusion}$ contains two types of complementary features but also allows the weight G to be fine-tuned in real-time as the input task changes. It is important to note that this “task-aware” capability is implicitly learned through the backpropagation of the CTC objective: the model spontaneously develops a mapping where G prioritizes the local branch during transient label prediction to capture precise boundaries, while shifting towards the global branch during blank-dominated segments. By optimizing this mapping end-to-end, the gate functions as a discriminative filter that effectively bridges the gap between pre-trained semantic representation and CTC alignment needs, without requiring explicit external task state inputs. This provides the model with high resource configuration flexibility at critical positions for CTC decoding (i.e., time points predicting non-Blank labels).

The core of this fusion mechanism lies in that the learning process of its gating weights is directly controlled by the constraints of the CTC loss function. This enables the gating mechanism to perform discriminative adjustments at the feature dimension level, automatically identifying which feature components need reinforced local alignment and which should focus on semantic representation. During training, gating parameters spontaneously adjust the dependence on local or global branches based on the specific function of each time step in the alignment path, thereby achieving a dynamic balance between alignment modeling and semantic modeling at the physical structural level. Unlike traditional fixed weighting or naive feature concatenation, this task-aware fusion mechanism forces the dual-branch structure to form a clear functional division under the CTC framework, which is key to the significant improvement in alignment stability and recognition performance achieved by this method.

### 2.4. Module Training and Optimization

The overall model uses CTC loss as the training objective, defined as:

(5) $$L_{CTC}=-logP\left(Y|Z_{fusion}\right)$$

where Y is the target text sequence, and $Z_{fusion}$ is the fused encoder output. During training, the local branch, global branch, and task-aware gating parameters jointly participate in end-to-end optimization.

Benefiting from the dual-branch structure being placed directly in the backpropagation loop of CTC alignment constraints, the two paths gradually evolve distinct temporal statistical representations during training iterations. The unique “Peaky Distribution” characteristic of the CTC algorithm actually plays an implicit guiding role: it drives the gating weights in Figure 3 to produce a significant polarity shift towards the local branch (Figure 2a), which is responsible for capturing short-term structures, at the transients where label prediction occurs, thereby precisely locking temporal boundaries. It must be pointed out that this functional differentiation does not rely on additional external supervision signals but originates from the deep coupling and synergistic induction between the physical structure design and the CTC training mechanism. In summary, while maintaining the standard CTC decoding framework unchanged, the proposed method successfully implements a structural reconstruction of the wav2vec 2.0 high-level temporal modeling paradigm, providing a feasible optimization path oriented towards the CTC alignment mechanism for end-to-end speech recognition models.

## 3. Results

### 3.1. Experimental Setup

The experiments were conducted on two representative public Mandarin speech recognition datasets. The first is AISHELL-1 [32], which contains approximately 178 h of Mandarin recordings from 400 speakers. Due to its standardized content and high annotation consistency, it is widely regarded as a benchmark for evaluating the basic acoustic modeling capabilities of E2E systems. The second is the ST-CMDS dataset [33], containing approximately 100,000 short speech sequences. Its key feature is the inclusion of diverse speaking styles from natural interaction scenarios, characterized by drastic speaking rate fluctuations and numerous acoustic variants, which effectively validates the model’s alignment robustness and generalization in complex acoustic conditions. In the experiments, all audio data were resampled to 16 kHz, and Global Variance Normalization (GVN) was applied to eliminate the interference of recording equipment gain differences.

For acoustic feature extraction and modeling, this study utilized the pre-trained model jonatasgrosman/wav2vec 2.0-large-xlsr-53-chinese-zh-cn for initial weights. This model, based on the wav2vec 2.0-Large architecture, was pre-trained on a massive multi-source Chinese corpus and possesses strong multi-scale feature representation capabilities. The baseline system employs the standard wav2vec 2.0 encoder followed by a CTC decoding layer. The proposed DBA-wav2vec 2.0 model implements structural reconstruction at the high level of the encoder: the local branch is constructed via multi-layer 1D depthwise separable convolutions (Conv1D) with a kernel size of 5 to capture fine-grained spectral evolution and transient boundary features; the global branch retains the self-attention mechanism to assist in semantic error correction. These two branches are integrated through a Task-Aware Gating mechanism, which adaptively adjusts the feature weights based on the confidence of the acoustic input. To ensure fairness and rigor, both the baseline and DBA-wav2vec 2.0 used identical experimental configurations for fine-tuning.

Regarding the training strategy, a two-stage end-to-end fine-tuning method was adopted using the AdamW optimizer [34] with an initial learning rate of $5\times 10^{-5}$. To ensure stability and prevent local optima, a Tri-stage Scheduler was employed, including a 10% linear warm-up, 40% constant high learning rate training, and 50% linear annealing. The experimental environment was based on NVIDIA A100 GPUs and the PyTorch framework (version 2.1.0) [35]. The primary evaluation metric is the Character Error Rate (CER). To ensure a fair and reproducible comparison, the Transformer-CTC and Conformer-CTC baselines were configured with a 12-layer encoder structure, a hidden dimension of 512, 8 attention heads, and a feed-forward dimension of 2048. The Conformer-CTC additionally employs depthwise separable convolutions with a kernel size of 15. All models were strictly aligned in terms of experimental conditions: no data augmentation (such as speed perturbation) or auxiliary loss functions were used during fine-tuning, and only Greedy Search was applied during the CTC decoding phase. This “minimalist” comparison aims to purely verify the efficiency of the temporal modeling structure on feature extraction. In the experimental reproduction and benchmark settings, we strictly followed the general evaluation criteria of open-source toolkits such as WeNet (version 3.0.0) [36].

### 3.2. Performance and Alignment Analysis

Table 1 shows the performance comparison across different architectures. Although the baseline wav2vec 2.0 already exhibits high performance, the proposed DBA-wav2vec 2.0 achieves relative CER reductions of 6.4% on AISHELL-1 and 7.4% on ST-CMDS with a minimal parameter increase of 4.1%.

To ensure that the observed performance gains are robust and not artifacts of random initialization variance during the fine-tuning of large-scale pre-trained models, we further evaluated the baseline and the proposed DBA-wav2vec 2.0 on the AISHELL-1 dataset across four different random seeds (including the one reported in Table 1). The baseline yielded an average CER of 6.58% (±0.05), whereas the proposed DBA-wav2vec 2.0 achieved a stable average CER of 6.17% (±0.03). The consistent absolute improvement significantly exceeds the standard deviation margin. Even the highest CER variation in the proposed model outperforms the lowest CER variation in the baseline, confirming that the architectural correction introduced by the dual-branch design robustly enhances acoustic-to-text alignment precision regardless of initialization variance.

**Figure 4:**
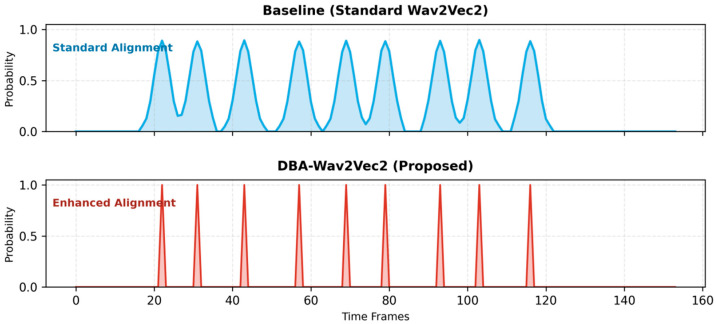
CTC probability distribution comparison between the Baseline and DBA-wav2vec 2.0.

To investigate the microscopic impact of the DBA structure on CTC alignment, we visualized the posterior probability distributions in Figure 4. The baseline displays “Broad Peaks” with high temporal uncertainty. In contrast, DBA-wav2vec 2.0 generates “Sharp Spikes” with highly focused energy, indicating that the local branch successfully corrects the global smoothing effect of the Transformer and enhances the precision of phoneme boundary detection.

### 3.3. Robustness Under Varying Speaking Rates

We further evaluated the robustness of the model on the ST-CMDS test set categorized by speaking rate (characters per second). The results are summarized in Table 2.

As shown in Table 2, the baseline performance degrades significantly in the “Fast” speech category (CER increasing to 10.45%) due to compressed phoneme durations. However, DBA-wav2vec 2.0 achieves a 15.3% relative improvement in this category, proving that the convolutional local branch effectively locks onto high-dynamic acoustic signals where standard self-attention fails.

## 4. Discussion

To isolate the key factors driving performance improvements and to evaluate the robustness of the proposed DBA-wav2vec 2.0, this discussion is structured into a hierarchical validation framework. We first provide a microscopic analysis of the model’s alignment stability to characterize the impact of our dual-branch design (Section 4.1). We then disentangle the architectural contributions by verifying the necessity of each component and fusion strategy (Section 4.2). Subsequently, we compare our method against generic adapter architectures to evaluate its generalization potential (Section 4.3). Finally, we perform a systematic exploration of the local modeling design space—including operator configuration and deployment strategy (Section 4.4)—to identify the optimal architectural configuration.

### 4.1. Analysis of Alignment Stability via Posterior Distributions

The performance gains can be interpreted through the lens of alignment stability. To objectively substantiate this claim, we extracted the posterior probability distributions across the test set to compute two quantitative metrics: Average Alignment Entropy and Average Peak Width. The statistical results are presented in Table 3.

As shown in Table 3, the baseline model exhibits a high average alignment entropy of 0.582 and an average peak width of 3.64 frames. This corresponds to the observation that the baseline model exhibits “Broad Peaks” in its posterior probability distribution, as visualized in Figure 4, indicating temporal uncertainty and blurred phoneme boundaries.

In contrast, the proposed architecture significantly reduces the alignment entropy to 0.247 and narrows the average peak width to 1.28 frames. Consistent with these metrics, DBA-wav2vec 2.0 generates “Sharp Spikes” with highly focused energy. This quantitative validation confirms that the local branch successfully compensates for the “attention smoothing effect” inherent in deep Transformer layers. By sharpening the peaks, the model reduces path search uncertainty in CTC, leading to more precise and stable acoustic-to-text alignment.

### 4.2. Effectiveness of the Dual-Branch Architecture

Ablation studies in Table 4 verify our working hypothesis that decoupling local and global streams is superior to unified modeling. To explicitly analyze the architectural contributions, we evaluated three variants: “Global-only” retains the self-attention path but removes the local convolutional branch; “Local-only” utilizes depthwise separable convolutions while excluding the global attention mechanism; and “Parallel Dual-branch” combines both streams via static summation rather than the proposed gating strategy.

The results indicate that the Task-Aware Gating mechanism allows for a “dynamic division of labor” that adapts to non-stationary acoustic signals more effectively than static addition.

Furthermore, we assessed the practical real-world applicability of the DBA-wav2vec 2.0 regarding computational overhead. Measured on a single A100 GPU with a batch size of 1 for 10-s audio segments, the DBA module introduces a marginal increase in computational cost as detailed in Table 5.

As shown in Table 5, the local convolutional branch introduces a marginal increase in computational overhead. Specifically, the FLOPs increase by approximately 6.9%, and the average inference latency increases by 3.4 ms. Given the significant CER reduction of 6.4% on the AISHELL-1 dataset, we consider this computational cost to be well-justified. The depthwise separable convolutions are highly efficient and effectively leverage modern GPU parallelization, ensuring that the architecture remains suitable for practical, real-time ASR systems.

### 4.3. Comparison with Generic Adapter Architectures

Beyond the internal component analysis, we further verified whether the performance gains stem from our specific architectural design or merely from increasing model parameters. We compared the proposed DBA against standard Conformer and Branchformer blocks acting as adapter heads on top of the same frozen wav2vec 2.0 backbone. To ensure a fair comparison, these blocks were configured to maintain a strictly comparable parameter budget (approximately 13.1 M added parameters) to our method.

As shown in Table 6, while the generic Conformer and Branchformer blocks provide modest performance gains (CER 6.44% and 6.38%, respectively), they are outperformed by the proposed DBA (6.15%). This confirms that simply stacking generic blocks continues to rely heavily on self-attention mechanisms, which may not effectively counteract the semantic smoothing of the pre-trained encoder. In contrast, the DBA’s explicit decoupling and task-aware gating offer a more suitable inductive bias for correcting alignment boundaries in CTC-based fine-tuning.

### 4.4. Interpretation of Operator Choice and Deployment Strategy

The sensitivity to kernel size (Table 7) and deployment depth (Table 8) provides insights into the structural requirements of CTC systems.

The optimal performance at Layer 24 confirms that the “semantic smoothing” accumulated through deep Transformer layers is the primary bottleneck for CTC alignment. Introducing the local branch at the top serves as a “re-alignment” mechanism that restores fine-grained acoustic boundaries before decoding.

## 5. Conclusions

In this paper, we addressed the core conflict between the “semantic smoothing effect” inherent in wav2vec 2.0 high-level representations and the “local discriminability” required by CTC decoding. We proposed DBA-wav2vec 2.0, a dual-branch attention speech recognition architecture specifically oriented toward the CTC alignment mechanism. Unlike traditional methods that focus on parameter tuning within a single path, this study reconstructed the temporal modeling logic of speech representation at the physical structural level. By explicitly constructing decoupled local and global parallel branches at the top of the encoder and introducing a dimension-wise task-aware gating mechanism, we achieved an adaptive fusion of feature flows optimized for CTC characteristics.

Empirical research on the AISHELL-1 and ST-CMDS datasets demonstrates that DBA-wav2vec 2.0 achieves relative CER reductions of 6.4% and 7.4%, respectively, while maintaining a nearly constant parameter scale. Notably, the model achieved a significant 15.3% performance leap in fast-speech scenarios. This work demonstrates that targeted structural correction at the encoder–decoder interface is a highly effective strategy for optimizing self-supervised representations for downstream ASR tasks. Future research will explore the integration of this architecture with other self-supervised frameworks and its performance in multi-speaker environments. Furthermore, while our dual-branch architecture is theoretically agnostic to the speech representation backbone, its synergy with other architectures (e.g., HuBERT or WavLM) and its generalization across different languages and decoding frameworks (e.g., RNNT or attention-based decoders) require further empirical verification. These aspects, alongside a detailed latency analysis for real-time streaming, represent the prioritized agenda for our future work, as the current study is primarily optimized for the CTC-based alignment bottleneck.

## Figures and Tables

**Table 1:** Comparison of performance and parameters across different architectures.

Model Architecture	Parameters (M)	Param. Incr. (%)	AISHELL-1 (CER%)	ST-CMDS (CER%)
Transformer-CTC	30.8	-	7.42	8.56
Conformer-CTC	47.5	-	6.95	8.02
Baseline (wav2vec 2.0)	315.4	-	6.57	7.53
**DBA-wav2vec 2.0**	**328.5**	**+4.1**	**6.15**	**6.97**

Note: All benchmark models are re-implemented in the same experimental environment for a fair comparison. Bold values indicate the best performance.

**Table 2:** CER performance comparison under different speaking rates on the ST-CMDS dataset.

Speaking Rate	Char/Sec	Ratio (%)	Baseline CER (%)	DBA-wav2vec 2.0 (%)	WERR (%)
Slow	<3.5	20.5	5.82	**5.61**	+3.6
Normal	3.5–5.5	58.2	7.15	**6.64**	+7.1
Fast	>5.5	21.3	10.45	**8.85**	+15.3
Overall	-	100	7.53	**6.97**	+7.4

Note: WERR denotes the relative Character Error Rate Reduction compared to the Baseline. Bold values indicate superior performance.

**Table 3:** Quantitative evaluation of CTC alignment stability on the AISHELL-1 test set.

Model Architecture	Average Alignment Entropy (↓)	Average Peak Width (Frames) (↓)
Baseline (wav2vec 2.0)	0.582	3.64
DBA-wav2vec 2.0	0.247	1.28

Note: (↓) indicates that lower values denote better alignment stability and precision. Peak width is measured in time frames (1 frame = 20 ms).

**Table 4:** Ablation study of DBA components and fusion strategies.

Model Structure	Global	Local	Fusion Strategy	AISHELL-1 (%)	ST-CMDS (%)
Baseline (wav2vec 2.0)	-	-	-	6.57	7.53
Global-only	✓	-	-	6.48	7.39
Local-only	-	✓	-	6.42	7.26
Parallel Dual-branch	✓	✓	Fixed Summation	6.34	7.18
**DBA-Wav2vec2**	✓	✓	**Task-Aware Gating**	**6.15**	**6.97**

Note: “Global” refers to the Self-Attention branch, and “Local” refers to the Depthwise Convolution branch. ✓ indicates the inclusion of a specific component. Bold values represent the best performance in each category.

**Table 5:** Computational complexity and inference latency analysis.

Model	Parameters (M)	FLOPs (G)	Latency (ms)	CER (%)
Baseline	315.4	120.5	45.2	6.57
DBA-Wav2vec2	328.5	128.8	48.6	6.15

Note: Inference latency is measured on a single NVIDIA A100 GPU with a batch size of 1 for 10-s audio segments.

**Table 6:** Performance comparison of different adapter architectures with comparable parameter budgets on AISHELL-1.

Model Configuration (Backbone: wav2vec 2.0)	Parameters (M)	Incr. (M)	CER (%)	Relative Reduction (%)
Baseline (None)	315.4	-	6.57	-
+Conformer Block (Adapter)	~328.5	~13.1	6.44	2.0
+Branchformer Block (Adapter)	~328.5	~13.1	6.38	2.9
+DBA (Proposed)	328.5	~13.1	6.15	6.4

Note: The Conformer and Branchformer adapter blocks are configured with comparable parameter budgets to our DBA module (~13.1 M added parameters). The backbone (wav2vec 2.0-Large) remains frozen for all configurations.

**Table 7:** Ablation of local branch operators.

Operator Type	Kernel Size	Pointwise Conv Layer	AISHELL-1 (%)	ST-CMDS (%)	Analysis
Standard Conv1D	5	-	6.49	7.32	High parameter load
Depthwise Conv	3	✓	6.45	7.29	Small receptive field
**Depthwise Conv**	**5**	**✓**	**6.42**	**7.26**	**Optimal config**
Depthwise Conv	7	✓	6.43	7.28	Redundant info
Removed Pointwise layer	5	-	6.51	7.40	No cross-channel mapping

Note: Bold values indicate the optimal settings selected for our final model.

**Table 8:** Impact of DBA module deployment location.

Deployment Location	AISHELL-1 (%)	ST-CMDS (%)	Analysis
Mid-layer (Layer 12)	6.45	7.35	Sufficient acoustic details in low-level
**Top-layer (Layer 24)**	**6.15**	**6.97**	**Compensates for semantic smoothing**
Multi-layer (12, 18, 24)	6.13	6.94	Diminishing marginal returns

Note: Bold values indicate the optimal configuration selected for the final model architecture.

## Data Availability

The datasets analyzed in this study are publicly available. The AISHELL-1 dataset can be found at http://www.openslr.org/33/ (accessed on 1 May 2025). The ST-CMDS dataset can be found at http://www.openslr.org/38/ (accessed on 1 May 2025).

## Abbreviations

The following abbreviations are used in this manuscript:

ASR	Automatic Speech Recognition
CTC	Connectionist Temporal Classification
CER	Character Error Rate
DBA	Dual-Branch Architecture
SSL	Self-Supervised Learning
